# Artificial Neural Network Model for Predicting Mechanical Strengths of Economical Ultra-High-Performance Concrete Containing Coarse Aggregates: Development and Parametric Analysis

**DOI:** 10.3390/ma17163908

**Published:** 2024-08-07

**Authors:** Ling Li, Yufei Gao, Xuan Dong, Yongping Han

**Affiliations:** 1 School of Civil Engineering and Transportation, Northeast Forestry University, No. 26 Hexing Road, Harbin 150040, China; gyfwudi11@163.com (Y.G.); dongxuan5858@163.com (X.D.); 2 Biochemical Engineering College, Beijing Union University, Fatou Xili District 3, Chaoyang District, Beijing 100023, China

**Keywords:** back-propagation artificial neural network, UHPC-CA, compressive and flexural strength, prediction, parametric study

## Abstract

Ultra-high-performance concrete with coarse aggregates (UHPC-CA) has the advantages of high strength, strong shrinkage resistance and a lower production cost, presenting a broad application prospect in civil engineering construction. In view of the difficulty in establishing a mathematical model to accurately predict the mechanical properties of UHPC-CA, the back-propagation artificial neural network (BP-ANN) method is used to fully consider the various influential factors of the compressive strength (CS) and flexural strength (FS) of UHPC-CA in this paper. By taking the content of cement (C), silica fume (SF), slag, fly ash (FA), coarse aggregate (CA), steel fiber, the water–binder ratio (*w/b*), the sand rate (SR), the cement type (CT), and the curing method (CM) as input variables, and the CS and FS of UHPC-CA as output objectives, the BP-ANN model with three layers has been well-trained, validated and tested with 220 experimental data in the studies published in the literature. Four evaluating indicators including the determination coefficient (*R*^2^), the root mean square error (*RMSE*), the mean absolute percentage error (*MAPE*), and the integral absolute error (*IAE*) were used to evaluate the prediction accuracy of the BP-ANN model. A parametric study for the various influential factors on the CS and FS of UHPC-CA was conducted using the BP-ANN model and the corresponding influential mechanisms were analyzed. Finally, the inclusion levels for the CA, steel fiber, and the dimensionless parameters of the *W/B* and sand rate were recommended to obtain the optimal strength of UHPC-CA.

## 1. Introduction

Ultra-high-performance concrete (UHPC) is an innovation of cementitious composite materials with superior mechanical performance and remarkable durability compared to conventional concrete, which has attracted extensive attention from scholars and engineers, and been expected to be popularized and applied in the civil engineering field [1]. However, the substantial need for cementitious materials in UHPC mixtures induces a high production cost and large early shrinkage in construction, restraining its further application in practical engineering. To alleviate these issues, the incorporation of a coarse aggregate (CA) with small particle sizes (less than 10 mm) in UHPC is considered as a promising approach that contributes to the formation of the equivalent skeleton to limit the initial micro-cracks in structures and can further improve the strength and stiffness of UHPC [2]. By introducing phosphogypsum aggregates to UHPC, Dong et al. proposed a novel green ultra-high-performance concrete. Base on the mechanical property experiment, it can be found that the introduction of a phosphogypsum aggregate in UHPC accelerated water migration and directly affected its hydration degree. And the mechanical performance of the UHPC containing phosphogypsum aggregates decreased slightly as the phosphogypsum aggregate content increased, but it was still capable of meeting the requirements of the applications [3].

The compressive strength (CS) is the most representative index for the mechanical properties of concrete. And for the commonly used load-bearing members, such as flexural members, compression-flexure members, and so on, the flexural strength (FS) is crucial to the mechanical properties of the components. Consequently, numerous studies have been conducted to investigate the two basic parameters of UHPC-CA to provide a basis for the design and calculation of UHPC-CA members and structures. Therein, the mixture design of UHPC-CA has the most intuitive effect on the mechanical properties of concrete. The influences of the water-to-binder ratio (*w/b*), steel fiber volume fraction, supplementary cementitious materials as well as coarse aggregate content on the CS and FS of UHPC-CA were widely studied [4,5,6,7]. Wu et al. [2] investigated the effects of coarse aggregate contents on the CS and FS of UHPC-CA through experiments. The test results showed a significant increase in CS and the elastic modulus of UHPC-CA after a moderate addition (no more than 30% by volume of mortar replacement) of coarse aggregate, while there was only a slight reduction in the FS and post-peak ductility. Similar results were found by Liu et al. [8] as well that when the replacement level of CA was less than 25%, the properties of UHPC were not impaired significantly. Moreover, the combined effect of CA and fiber properties on UHPC was investigated. Three types of steel micro-fibers (i.e., smooth, spiral and hooked fibers) with a diameter of 0.2 mm, and one type of steel macro-fiber with a diameter of 0.6 mm were investigated. Therein, the tensile strengths of the smooth, spiral, and hooked steel micro-fibers and steel macro-fiber were 2940 MPa, 2860 MPa, 2940 MPa, and 1890 MPa, respectively. It was found that the inclusion of CA reduced the bonding strength and utilization efficiency of fibers, especially for deformed ones, due to the severe interlock between fibers and CA. Ma et al. [9] reported that UHPC with a coarse aggregate could be fluidized more easily and decreased the mixing time. Aghayan et al. [10] investigated the mechanical properties of roller-compacted concrete by replacing natural aggregate with ceramic waste aggregate. The experimental results showed that after replacing 15% natural aggregates with ceramic waste aggregates, the 90 d CS and FS were increased by 14 and 20%, respectively. Yoo and Banthia [11] reviewed the effect of mineral admixtures on the mechanical performance of UHPC. It can be concluded that the use of silica fume (SF) accelerated the hydration process of UHPC, thus improving the strength of UHPC. As the SF content increased up to 30%, the bond strength and pullout energy of fibers were remarkably increased. Moreover, the mechanical properties of UHPC were improved by using the deformed steel fibers compared to the use of straight steel fibers. In addition, replacing the SF with fly ash (FA) and ground granulated blast furnace slag (GGBFS), which can also be used as a fine silica source for UHPC, can delay the hydration process and have a positive effect on the flexural strength and toughness of UHPC. Zhang et al. [12] reported that heat treatment on UHPC resulted in an acceleration of the hydration process and an increased density leading to the ultra-high strength of UHPC, while the specimens with a lower curing temperature generally required a longer curing period to achieve similar strengths to those achieved with heat treating.

In conclusion, the influential factors on the strength of UHPC are various and the influential mechanisms of each factor are complex and even interrelated. Based on the experimental results, many researchers proposed empirical formulas for the compressive strength and flexural strength of UHPC, as listed in Table 1. It can be seen that the main calculation parameters in the formulas include the binder–water ratio (B/W), the volume of fraction and the geometric characteristics of steel fibers, and so on. And, the relationships between the predicted strength and the independent variables are mainly linear or quadratic functions. Due to the different research focus and the experimental conditions designed by each researcher, the influencing factors considered in each calculation formula are not comprehensive, and the generalizability of the predicted formulas is relatively poor. Furthermore, there are few empirical formulas to consider the influence of a coarse aggregate on the material properties in UHPC-CA. Therefore, it is challenging to derive the generalizable prediction methods of the strength of UHPC-CA from the limited experimental studies.

In recent years, with the development of Artificial Intelligence (AI), an AI technique of an artificial neural network (ANN) has exhibited great potential for solving various problems in civil engineering. Solhmirzaeisuch et al. [18] analyzed the failure mode and shear capacity of ultra-high-performance concrete beams reinforced with steel bars by using ANN models. Huang et al. [19] calculated the bond behavior between FRP bars and concrete at high temperatures by using a back-propagation ANN paradigm. Yan et al. [20] investigated the hysteretic performance of a composite-reinforced concrete beam, which was formed of a steel beam and a concrete slab, through an innovative ANN model. Fan et al. [21] proposed an approach for the precise design and characteristic prediction of UHPC by employing a Genetic Algorithm-based ANN technique. Khafajeh et al. [22] proposed an ANN model to evaluate longitudinal and transverse cracks in asphalt pavements. These classical cases can effectively verify the feasibility of the application of AI techniques in the civil engineering field. In the ANN model, all the possible interactions between the independent variables and their effects on the outputs can be automatically evaluated with highly accurate predictions [23,24,25,26,27]. Table 2 lists the information on the proposed ANN models for predicting the mechanical properties of various types of concrete, which demonstrates that the ANN methods can provide relatively accurate prediction results at a lower cost. For the learning techniques employed in training networks, back-propagation (BP) is considered to be one of the most widely used methods due to its computational advantages of strong nonlinear mapping, self-learning, and adaptive and generalized fault-tolerant abilities [19]. Although there are several studies on the ANN models for predicting the strength of UHPC, the input variables considered in each model may be not comprehensive. Moreover, with the introduction of a CA, the variation of regular UHPC strength with other mixtures may be changed. Therefore, in this paper, the content of a coarse aggregate was used as an input variable in an ANN model, which can more exactly capture the influences of the addition of a CA on the interactions between the independent variables and its effect on the outputs.

The aim of this paper is to propose a new BP-ANN model for the accurate prediction of the CS and FS of UHPC-CA that considers the various influential parameters as comprehensively as possible, while being convenient for the applications of practical engineering. A total of 220 experimental data were gathered to establish the BP artificial neural network models and a description of the development procedure was provided here. Ten variations including the content of cement (C), silica fume (SF), slag, fly ash (FA), a coarse aggregate (CA), steel fibers, the water–binder ratio (*w/b*), sand rate (SR), cement type (CT), and curing method (CM) were designated as input variables, and the compressive strength (CS) and flexural strength (FS) were specified as output variables. The universality and accuracy of the BP-ANN models were verified by comparing with the experimental data and the evaluating indicators. Finally, a parametric study was carried out to evaluate the influence of crucial variables on the CS and FS of UHPC-CA, and the influential mechanisms were analyzed systematically. 

## 2. Dataset Establishment

**Figure 1:**
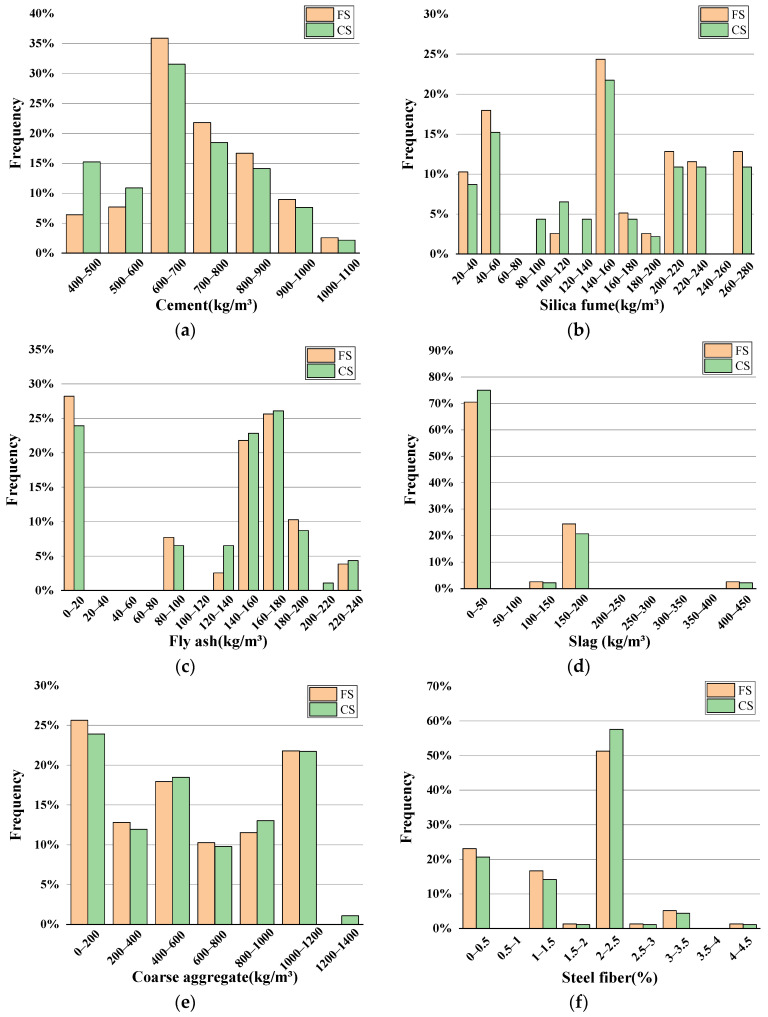

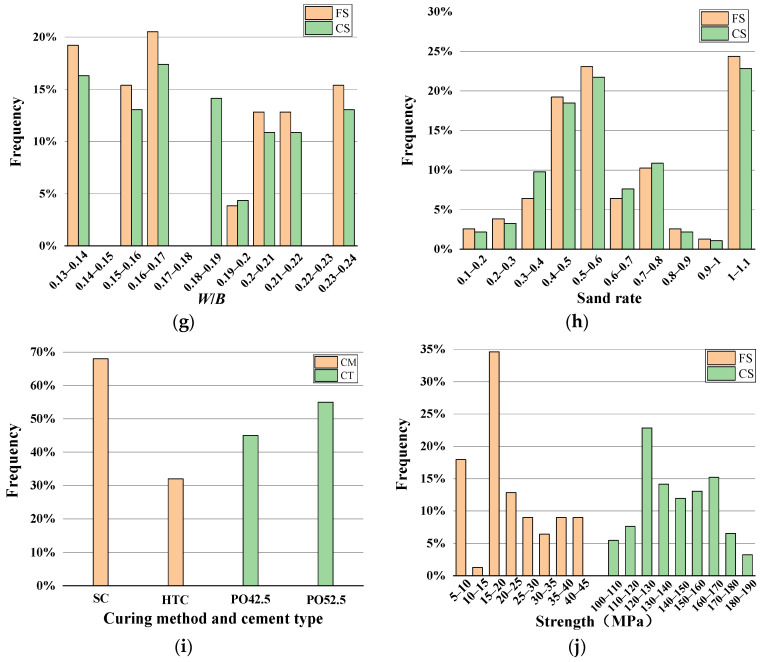
Frequency histogram of input and output variations: (**a**) Cement; (**b**) Silica fume; (**c**) Fly ash; (**d**) Slag; (**e**) Coarse aggregate; (**f**) Steel fiber; (**g**) *W/B*; (**h**) Sand rate; (**i**) Curing method and cement type; (**j**) Strength.

To investigate the effect of different factors on the strength of UHPC-CA, 220 experimental data were collected from the available studies and two datasets of the compressive strength (CS) and flexural strength (FS) of UHPC-CA were established [1,2,5,34,35,36,37,38,39,40,41,42,43,44,45], as shown in Appendix A. Ten variables were selected as input variables, including cement (C), silica fume (SF), slag, fly ash (FA), coarse aggregate (CA), steel fibers, the water–binder ratio (*w/b*), sand rate (SR), cement type (CT), and the curing method (CM). Among the collected datasets, two types of ordinary Portland cements, namely P.O. 42.5 and P.O. 52.5, were selected and assigned the values of 1 and 2 as input parameters. And, two types of curing methods were taken into account, namely standard curing (SC) and high-temperature curing (HTC) (60~90 °C), corresponding to the input variables of 1 and 2, respectively. Moreover, in order to eliminate the influence of coarse aggregate size on the strength of UHPC, the particle sizes of the coarse aggregate selected in this paper ranged from 5 to 10 mm. Table 3 and Table 4 show the statistical parameters of the datasets for the compressive and flexural strengths of UHPC-CA, respectively. It should be noted that the Standard Deviation (SD) of some parameters was relatively large. This is because the collected data were widely distributed and disperse, which was beneficial to build a wide application range for an ANN model. The dimensions of the test specimens for CS and FS were 100 × 100 × 100 mm and 100 × 100 × 400 mm. It can be seen that the ranges of compressive and flexural strengths of UHPC-CA are from 100 to 189.4 MPa and 9.36 to 43 MPa, respectively. Figure 1 shows the frequency histograms of the input and output variables. The mix design parameters in the datasets are all within the common ranges, such as the water–binder ratio being less than 0.25 to ensure the high compressive strength of the UHPC. 

**Figure 2:**
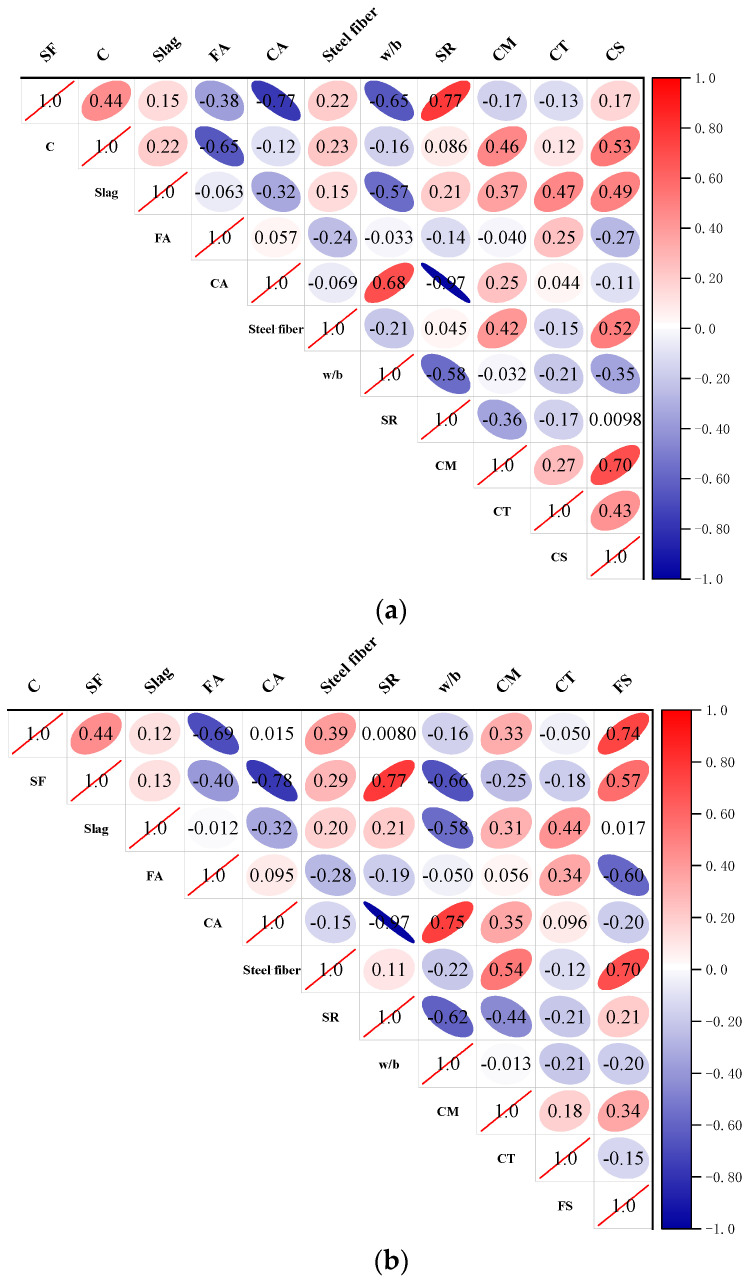
Pearson correlation matrix: (**a**) Compressive Strength; (**b**) Flexural Strength.

Figure 2 depicts the correlations of all the input and output variables by using the Pearson correlation matrix. The Pearson coefficient, which can measure the strength of the linear relationship between the two variables, is in the range of −1 to +1. A value of zero implies that there is no link between the variables, while the values close to 1 or −1 signify a stronger positive or negative relationship between the variables. 

## 3. Methodology

### 3.1. BP Artificial Neural Network

An Artificial neural network (ANN) is an information processing system for large-scale distributed parallel information processing [46] and has been widely applied in curve fitting problems. In the ANN architecture, the numerous neurons are distributed into three or more layers including an input layer, one or more hidden layers, and an output layer. All the neurons in each layer are connected to the neurons in the next layer by the network weights and biases. The back-propagation algorithm (BP) is one of the simplest and most frequently used training methods for the ANN model, in which the learning process consists of forward propagation and error back-propagation. In forward propagation, the input values are multiplied with the specific weights and then summed up with the bias. The sum is processed by the predefined activation functions and transferred to the next layer, as expressed in Equation (1). In this model, sigmoid-type activation functions are adopted in each layer. If the predicted results of the output layer do not reach the expected values, the process reverses into back-propagation, wherein the weights and biases of each layer are modified constantly to obtain a desired error.

(1) $$y_{j}=f(net_{j})=f(\sum_{i=1}^{n}w_{ij}x_{i}+b_{j})$$

where *x_i_* and *y_j_* are the inputs and outputs of the *j*th neuron; *w_ij_* is the weight between the *i*th and *j*th neuron; *b_j_* is the bias of the *j*th neuron; *n* is the number of neurons; and *f* is the activation function.

**Figure 3:**
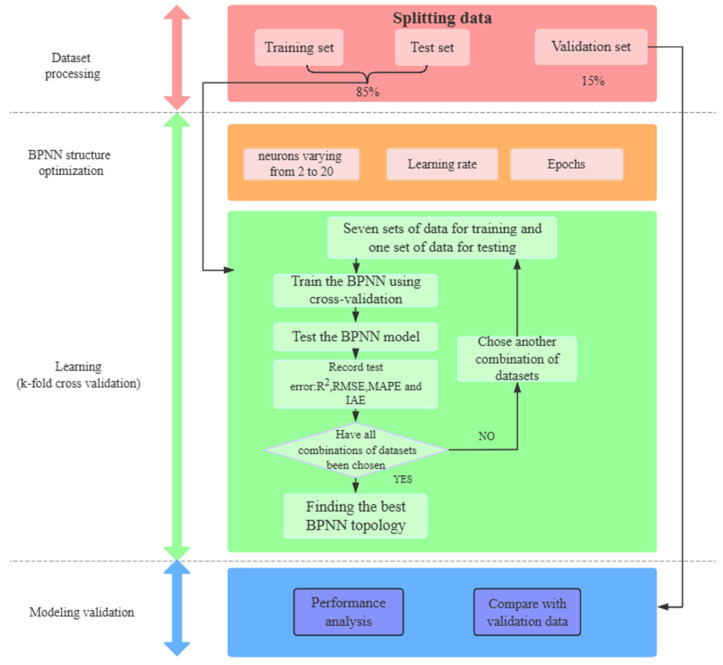
Flowchart explaining different steps in developing the BP-ANN model.

Based on the above theory, the back-propagation algorithm neural network process for predicting the strength of UHPC-CA is depicted in Figure 3. In the first step, all the collected experimental results of the CS and FS of UHPC-CA were randomly split into three parts, in which 75% of the data were used for training to establish the BP-ANN model and obtain the optimal weights and biases, while 10% of the data were selected for testing to verify the performance of the developed BP-ANN model, and the remaining 15% were used for validation to prevent overfitting the BP-ANN model. Before the training of the BP-ANN model, the input data should be linearly normalized since the sigmoid activation function is sensitive to variations between 0 and 1. This linear transformation preserved all the numerical relationships of the initial database. 

In the second step, a three-layer BP-ANN model was built using the training dataset. One hidden layer was configured in the present neural network and the number of the corresponding neurons was of great importance to the accuracy of the BP-ANN model. Since there are no general rules to exactly determine the number of neurons in the hidden layer, it was assumed to range from 2 to 20 in this study. Moreover, the number of epochs, which represents the times the entire dataset goes through the learning algorithm, is an essential hyperparameter that can significantly affect the model performance. To obtain an optimal BP-ANN structure, the k-fold validation process was selected to determine the above relevant parameters. In this model, k was set to 8 following the references provided by Hastie et al. [47] and the root mean square error (RMSE) measurements of the 8-fold validation were averaged to evaluate the accuracy. Based on the optimal BP-ANN model, the correlation and the corresponding indices of accuracy between the predicted and experimental outputs for both the training and testing datasets were compared and analyzed to further prove the accuracy of the model. 

In the third step, the optimized neural network model was used to calculate the CS and FS of the validation dataset and to compare the predicted values with the original results to further verify the performance of the BP-ANN model.

### 3.2. Evaluating Indicator 

Four indices of accuracy were calculated to evaluate the accuracy of the proposed BP-ANN model for predicting the CS and FS of UHPC-CA, namely, the determination coefficient (*R*^2^), the root mean square error (*RMSE*), the mean absolute percentage error (*MAPE*), and the integral absolute error (*IAE*). The *R*^2^ represents the degrees of correlation between the predicted values of the model and the experimental true values. The *RMSE* indicates the deviation between the predicted values and the experimental true values. The *MAPE* is a percentage of the mean value of the absolute error, which can reflect the actual scenario of the predicted value error. The *IAE* is the integral formula of a function of the deviation between the expected output of the system and the actual output or main feedback signal. The calculation formulas for each index are shown in Equations (2)–(5):

(2) $$R^{2}=1-\frac{\sum_{k=1}^{n}{\left(p_{k}-t_{k}\right)}^{2}}{\sum_{k=1}^{n}{\left(t_{k}-t\right)}^{2}}$$

(3) $$RMSE=\sqrt{\frac{1}{n}\sum_{k=1}^{n}{\left(p_{k}-t_{k}\right)}^{2}}$$

(4) $$MAPE=\left(\frac{1}{n}\sum_{k=1}^{n}\frac{\left|p_{k}-t_{k}\right|}{t_{k}}\right)$$

(5) $$IAE=\frac{\sum_{k=1}^{n}\left|p_{k}-t_{k}\right|}{\sum_{k=1}^{n}t_{k}}\times 100\%$$

where *n* is the total number of data in the training and testing datasets; *p_k_* and *t_k_* are the predicted and experimental values of the *k*th data, and *t* is the average of the experimental values. A prediction model is regarded with a higher precision when the *R*^2^ is closer to 1, and the *RMSE*, *MAPE* and *IAE* are closer to 0.

### 3.3. Determination of the Neurons in the Hidden Layer

**Figure 4:**
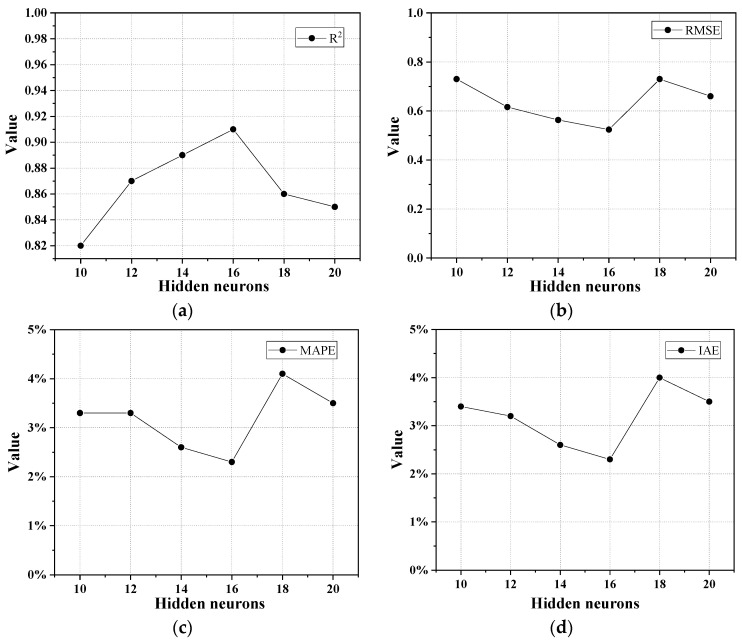
Evaluation indexes of the CS prediction model with different numbers of neurons in the hidden layer: (**a**) *R*^2^; (**b**) *RMSE*; (**c**) *MAPE*; (**d**) *IAE*.

**Figure 5:**
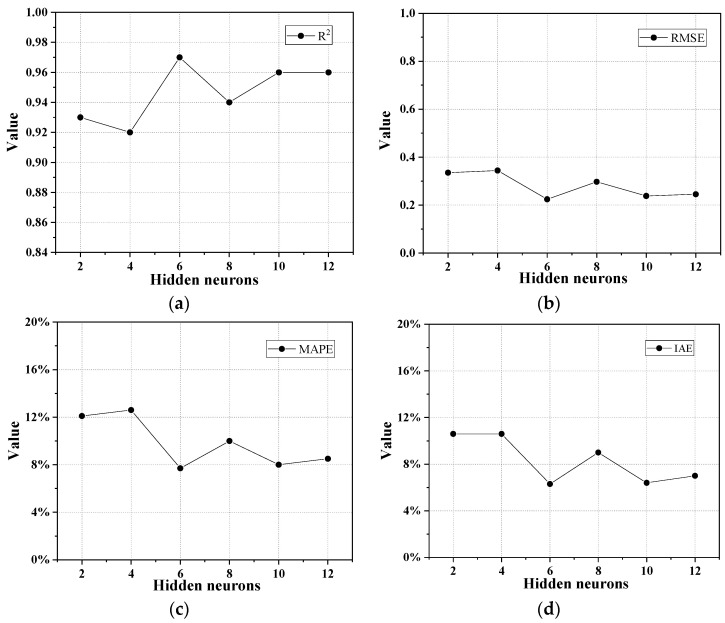
Evaluation indexes of the FS prediction model with different numbers of neurons in the hidden layer: (**a**) *R*^2^; (**b**) *RMSE*; (**c**) *MAPE*; (**d**) *IAE*.

**Figure 6:**
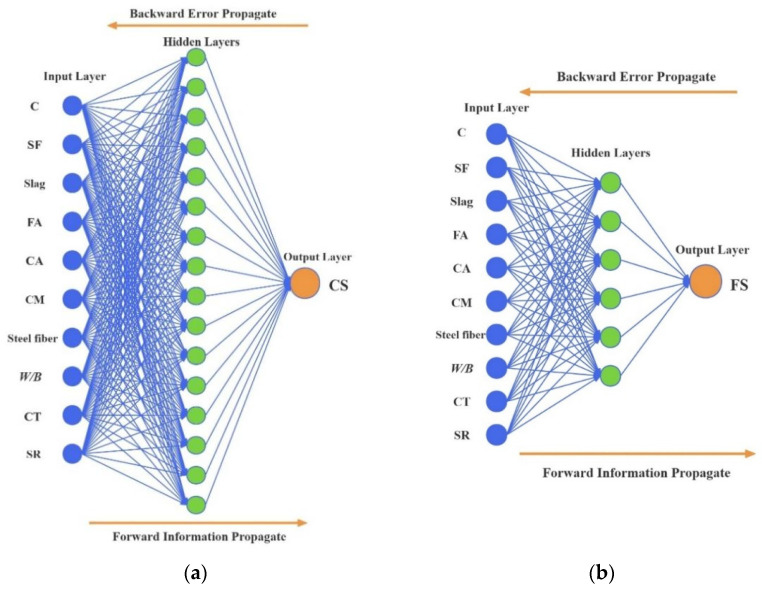
The architecture of the proposed BP-ANN model: (**a**) compressive strength; (**b**) flexural strength.

The number of neurons in the hidden layer of the BP neural network is directly related to its prediction accuracy. An excessive number of neurons may expand the training time and over-fit the training data, which may lead to a reduction in the calculation efficiency. However, if the number of neurons is not enough, insufficient information may result in a decrease in computational accuracy. Currently, the determination of the optimal number of neurons is mostly based on experience and trial. Through repeated trial and error, the numbers of the neurons in the hidden layer for predicting the CS and FS of UHPC-CA were selected from 10 to 20 and from 2 to 12, respectively. Figure 4 and Figure 5 show the evaluation indexes of the prediction model for CS and FS with different numbers of neurons in the hidden layer. It can be observed that when the numbers of neurons in the hidden layer for the CS and FS prediction model were 16 and 6, the evaluated indices of the *R*^2^ reached the maximum value, while the values of the *MSE*, *MAPE*, and *IAE* reached the minimum, which indicated that the overall predicted effect was the best. Therefore, the architecture of the proposed BP-ANN model is depicted in Figure 6.

### 3.4. BP-ANN Performance

**Figure 7:**
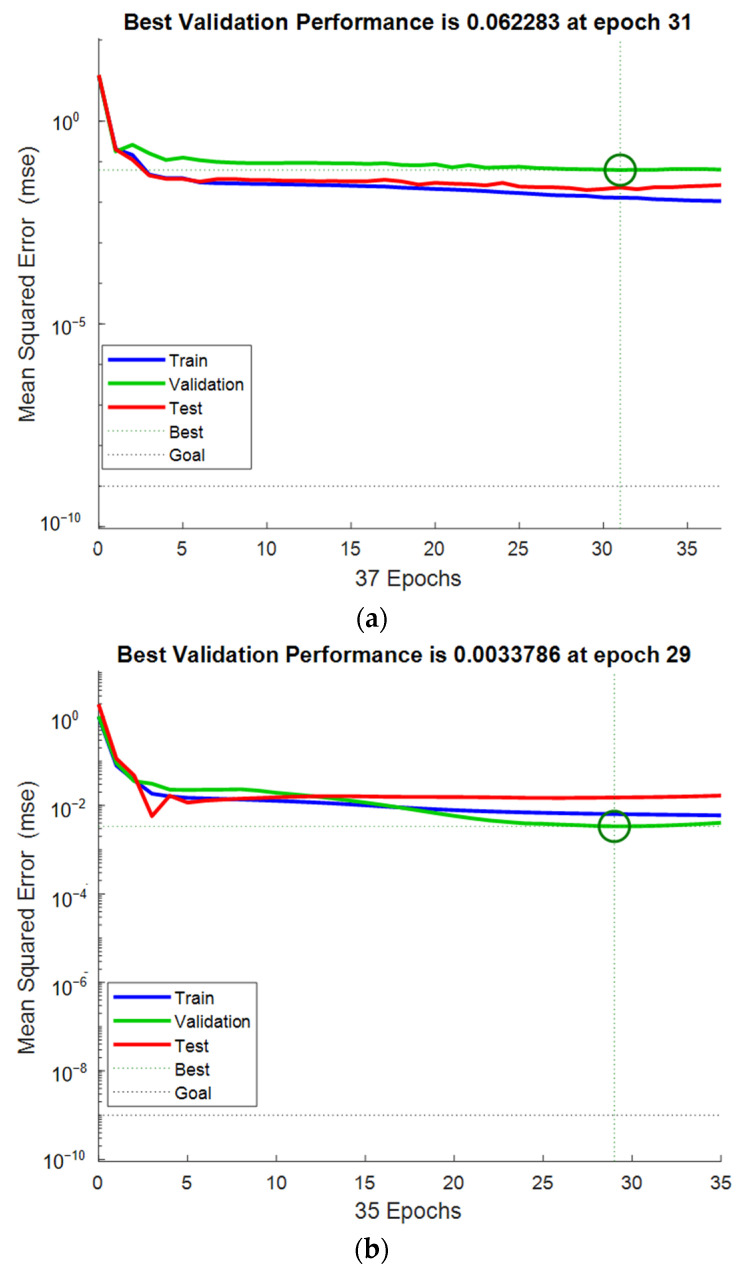
The performance of the proposed networks: (**a**) compressive strength; (**b**) flexural strength.

**Figure 8:**
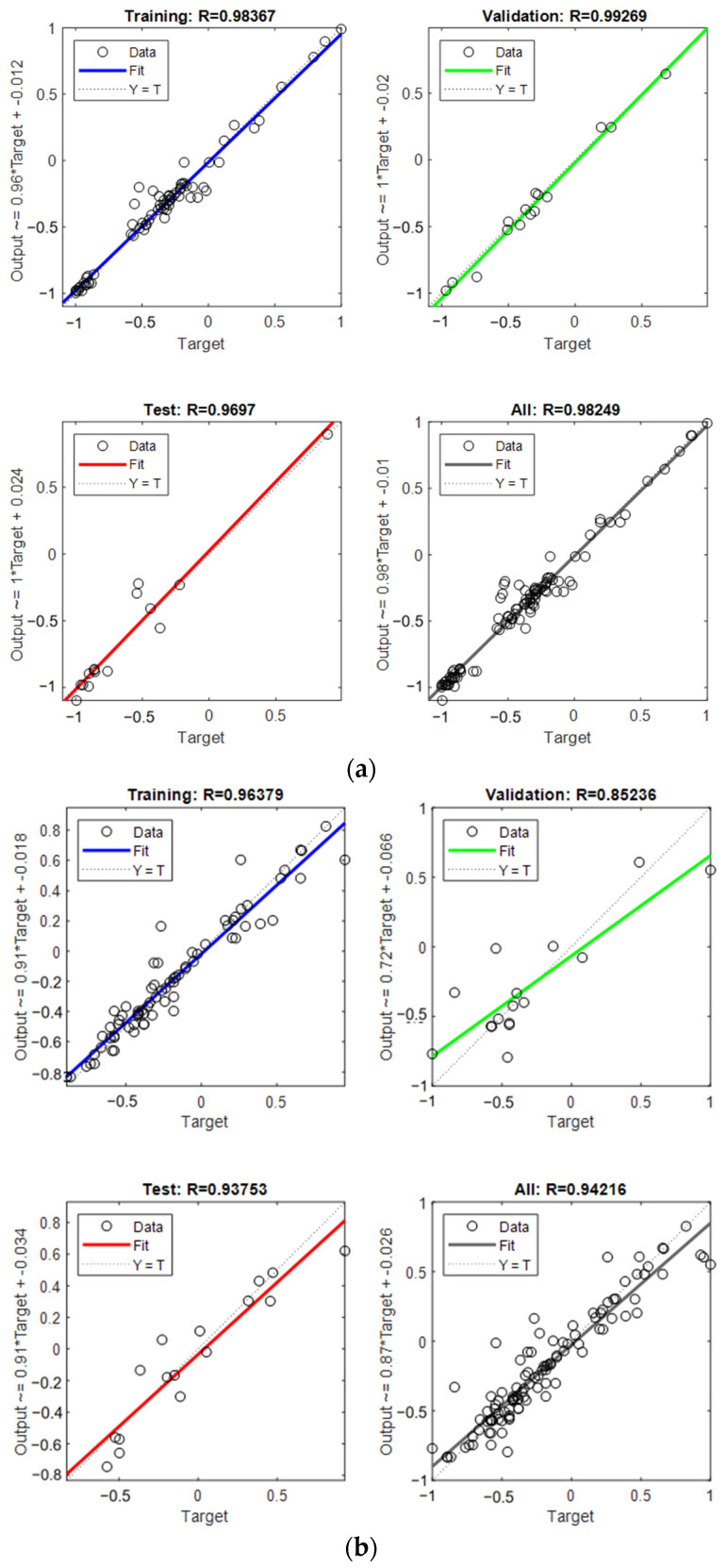
The regression of the proposed networks: (**a**) compressive strength; (**b**) flexural strength.

**Figure 9:**
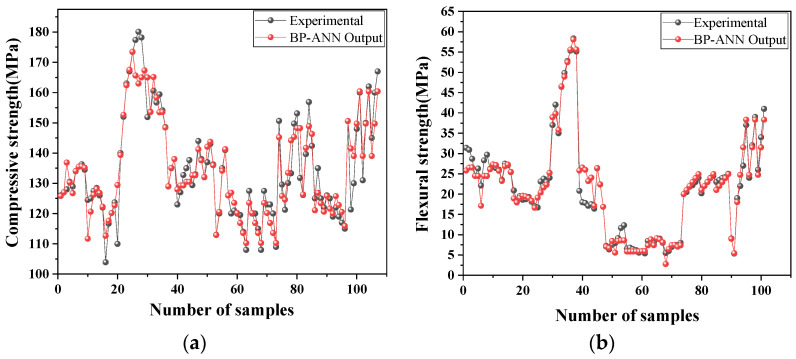
The comparison of the predicted results and the experimental data: (**a**) compressive strength; (**b**) flexural strength.

The performance of the network in estimating the CS and FS of UHPC-CA is shown in Figure 7. The best validation performance can be characterized by the minimum value of the MSE. It can be seen that for the CS of UHPC-CA, the MSE was 0.062 at the 31st epoch, while that for the FS of UHPC-CA was 0.003 at the 29th epoch, as depicted at the green circles in Figure 7. The correlations between the targets (experimental results) and the outputs (predicted results) of the proposed BP-ANN model are represented by the correlation coefficient R, as shown in Figure 8. It can be observed that the correlation coefficients of R for the training dataset, the testing dataset and the validation dataset are all close to 1, which verified the proposed BP-ANN model with high accuracy. As shown in Figure 9, the comparison of the predicted results and experimental data shows that the model has high accuracy.

## 4. Results and Discussions

Based on the proposed BP-ANN model, the variations in the CS and FS of UHPC-CA with various influencing factors can be obtained, and a systematic parameter study is conducted as discussed below. It should be noted that when one factor is studied, the other factors are kept constant.

### 4.1. The Influence of Coarse Aggregate Content

**Figure 10:**
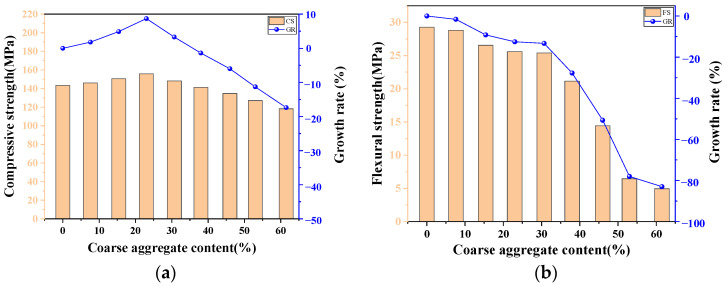
The influence of coarse aggregate content on (**a**) compressive strength and (**b**) flexural strength.

UHPC is well known for its dense matrix resulting in excellent mechanical properties. Therefore, it is intuitively believed that the addition of coarse aggregates will reduce the strength of UHPC. However, recent research indicated that an appropriate addition of coarse aggregates may have a positive effect. Based on the ANN predicted model, the variations of the CS and FS of UHPC-CA were obtained as the coarse aggregate contents ranged from 0 to 60%, as shown in Figure 10. 

It can be observed that the CS of UHPC-CA exhibited a trend of first increasing and then decreasing with the increase in the coarse aggregate content. For the UHPC without coarse aggregates, the compressive strength of the cube was 143.38 MPa, while it reached a maximum of 155.83 MPa when the coarse aggregate content increased to 23%. Then, the CS of UHPC-CA turned to decrease with the increase in coarse aggregate content. When the coarse aggregate content exceeded 23%, the CS of UHPC-CA was smaller than that of the unmixed coarse aggregate. Moreover, Li et al. [48] reported that with an increase in the CA content, the elastic modulus of UHPC-CA improved obviously as well. This can be interpreted from two aspects. Firstly, the coarse aggregates have high compressive strength and rough surfaces, which may enhance the bonding properties of the interface transition zones (ITZs) between the cementitious matrix and the coarse aggregates. Furthermore, introducing coarse aggregates can form an equivalent skeleton due to the aggregates interlocking, thus improving the overall compression performance. However, when the coarse aggregate content exceeds a certain threshold, the workability of UHPC-CA becomes worse, resulting in a severe agglomeration of fibers and increasing the internal defects in the matrix, which is unfavorable to the compressive properties of UHPC-CA. In addition, the bonding of ITZs is weakened due to the insufficient wrapping of slurry to the excessive coarse aggregates, which can also reduce the compressive strength of UHPC-CA.

**Figure 11:**
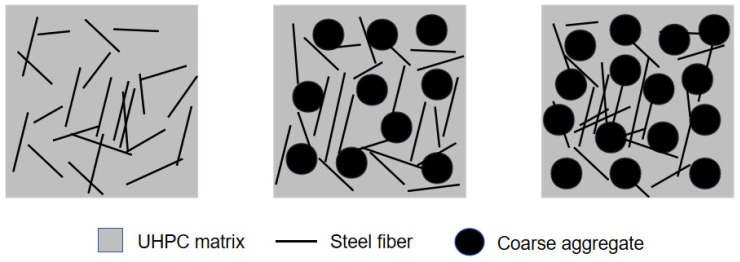
Schematic illustration of the agglomeration of steel fibers.

Figure 10b shows that there are no significant variations in the FS of UHPC-CA as the coarse aggregate content ranged from 0 to 30%. This is because when the additions of coarse aggregates are less, the matrix of UHPC-CA is relatively dense and the bonding between the fibers and the matrix is still effective. Meanwhile, the interlocking effect of the coarse aggregates may prevent the development of flexural-tensile cracks, which can offset the negative impact on the declined utilization of fiber caused by the addition of coarse aggregates to a certain extent, as shown in Figure 11. However, an excessive amount of coarse aggregates may lead to an accumulation of the internal defects in UHPC-CA and reduce the uniform distribution of steel fibers. Therefore, a sharp decline in the FS of UHPC-CA can be observed with the increase in coarse aggregates, as shown in Figure 10b. 

It should be noted that the strength of UHPC may be decreased with an increase in CA size, theoretically. This can be illustrated in the weaker interfacial transition zone (ITZ) between the aggregate and the paste, and more stress concentration at the contact points between aggregates can be obtained as the CA sizes increase. However, previous studies [34] have shown that the degree of decrease in UHPC strength affected by CA size is rather limited. The optimal CA size ranges from 5 mm to 10 mm. In conclusion, it can be seen that the admixture of coarse aggregates in UHPC should be controlled, and the appropriate mass content is between 15 and 30% with optimal sizes of coarse aggregates. 

### 4.2. The Influence of the Water–Binder Ratio

**Figure 12:**
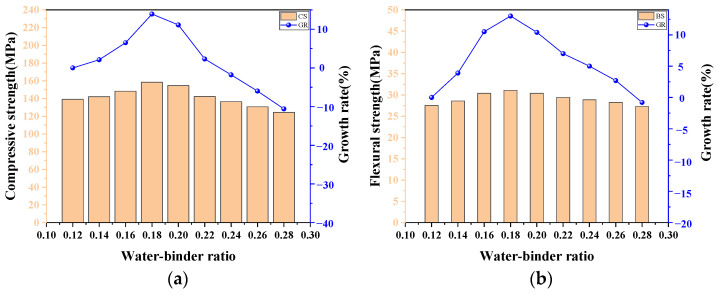
The influence of the water–binder ratio on the (**a**) compressive strength and the (**b**) flexural strength.

The water–binder ratio *W/B* is a primary factor that affects the mechanical properties of UHPC-CA since it is closely related to the hydration reaction of cementitious materials [49]. Figure 12a shows the relationships between the CS of UHPC-CA and the water–binder ratios. It can be seen that the CS of UHPC-CA reached a maximum value of 158.39 MPa when the *W/B* was 0.18. As the *W/B* increased from 0.12 to 0.18, the CS exhibited an increasing trend and was increased by 13.9%, while as the *W/B* increased from 0.18 to 0.28, the CS exhibited a decreasing trend and was reduced by 21.5%. This variation tendency was consistent with the test results reported by Shi [38]. The influential mechanisms can be illustrated as follows. The strength of UHPC-CA mainly stems from the hydration reaction and the pozzolanic effect between the cementing material and water to form a dense microstructure of concrete. With the increase in the water–binder ratio, the porosity of the UHPC-CA will increase due to the larger evaporation of residual water after hardening, leading to a decrease in the effective stress area and thus decreasing the compressive strength of the UHPC-CA. Therefore, theoretically, the lower the water–binder ratio, the denser the matrix and the higher the strength of UHPC-CA. Whereas in practical situations, an excessively low water–binder ratio may result in a decline in slurry flowability and an insufficient hydration reaction of certain cementing materials, which will increase the non-uniformity of UHPC-CA and decrease the CS of UHPC-CA in contrast. The regular variations in the FS of UHPC-CA with the water–binder ratio are similar to those of the CS, but the ranges of change in FS are gentle. As shown in Figure 12, the FS of UHPC-CA is between 27 and 31 MPa as the water–binder ratio varies from 0.12 to 0.28.

### 4.3. The Influence of Steel Fiber Volume Fraction

**Figure 13:**
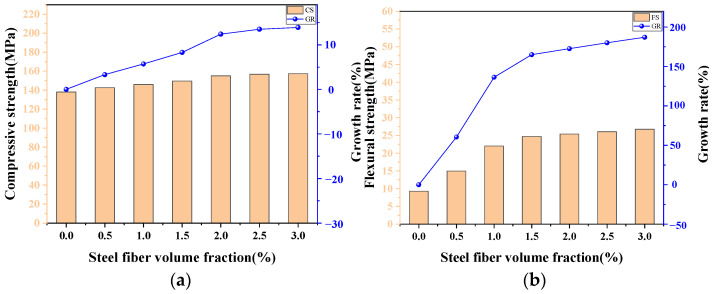
The influence of steel fiber volume fraction on the (**a**) compressive strength and the (**b**) flexural strength.

Fiber reinforcement plays an important role in the incensement of mechanical properties and the toughness of UHPC-CA in avoiding explosive behavior at failure [50], wherein steel fiber is the most commonly used fiber type. Figure 13 depicts the variations in the CS and FS of UHPC-CA with steel fiber volume fractions obtained from the proposed ANN model. It can be seen that the incorporation of steel fibers is beneficial to both the CS and the FS of UHPC-CA. Li et al. [7] employed three-point bending tests to measure the flexural performance of UHPC-CA, and similar results were obtained demonstrating that the FS and energy absorption capability of UHPC-CA increased with the increase in the steel fiber content, whereas the first crack stress was insensitive to the steel fiber content. The reinforcing mechanism is that the steel fibers can effectively restrain the initiation and development of micro-cracks and macro-cracks in UHPC-CA under tensile force and compression, which is called the fiber bridging effect. However, the effect degrees on these two strengths are different, in which the relative change in FS is more obvious. As the steel fiber volume fraction increased from 0 to 3%, the CS of UHPC-CA was improved by 13.9%, while the FS of UHPC-CA was increased by 3.6 times, as shown in Figure 13. An explanation for this may be that the CS of UHPC-CA is mainly determined by the strength of the matrix and the promotive effect of steel fiber is limited. Moreover, in the different ranges of steel fiber volume fractions, the increase rates of the CS and the FS of UHPC-CA are not the same. When the steel fiber volume fraction increased from 0 to 1% and 1% to 3%, respectively, increases of 6% and 4% were obtained for the CS and the FS of UHPC-CA was increased by 2.4 times and 1.21 times. The reason for this is that the workability of UHPC-CA may be worse due to the fiber agglomeration for a certain value of steel fiber volume fraction, and the defects in the microstructure will increase, leading to a decrease in the CS and FS of UHPC-CA. These indicate that the optimal steel fiber volume fraction for UHPC-CA is about 1.5~2%, which can achieve excellent mechanical properties in the concrete and better economic benefits. 

### 4.4. The Influence of Supplementary Cementitious Materials

**Figure 14:**
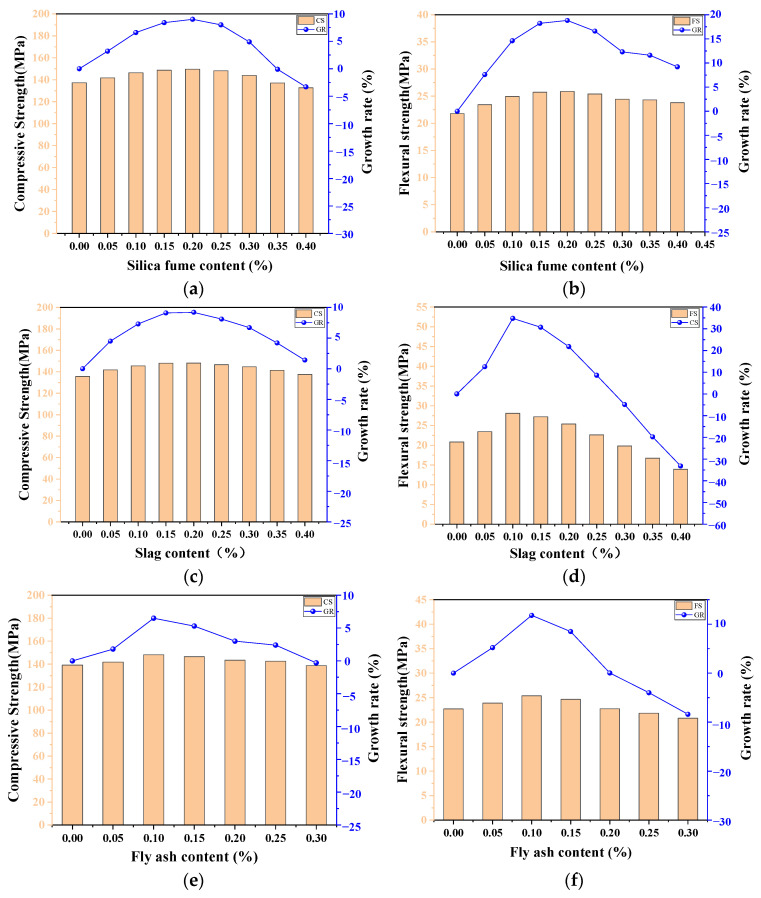
The influence of supplementary cementitious materials: (**a**) The effect of SF on compressive strength; (**b**) The effect of SF on flexural strength; (**c**) The effect of slag on compressive strength; (**d**) Effect of slag on flexural strength; (**e**) The effect of FA on compressive strength; (**f**) The effect of FA on flexural strength.

In the preparation of concrete, the most favorable hydration product to ensure a dense microstructure is calcium silicate hydrate (C–S–H) gel. It is reported that the usage of mineral admixtures can enhance the chemical reaction due to the increased surface area and maximize the formation of C–S–H with a lower water–binder ratio [51]. Therefore, it is necessary to replace part of the cement in UHPC-CA with supplementary cementitious materials, in which industrial by-products such as silica fume (SF), fly ash (FA), and slag are commonly used with the aim of obtaining a high density and better fluidity, while reducing the costs and environmental burden. According to the ANN model, the effects of slag content, SF content, and FA content on the CS and FS of UHPC-CA can be predicted as shown in Figure 14. It can be seen that the addition of supplementary cementitious materials is beneficial to the strength of UHPC-CA within an appropriate range of the mass ratio between the admixtures and cements. Meanwhile, the improvement in the CS of UHPC-CA is more significant compared to that of the FS. This favorable influence can be explained by three aspects. Firstly, the supplementary cementitious materials with a finer size act as a filler material to fill the voids between particles and the pores in the matrix, which is called the micro-aggregate effect, making the microstructure denser. Secondly, the fine spherical particles of supplementary cementitious materials can be treated as a lubricant, improving the workability of UHPC-CA. Lastly, the additional C–S–H produced by the secondary pozzolanic reaction between the supplementary cementitious materials and Ca(OH)_2_ may increase the strength of the matrix and play a further filling role, thus enhancing the strength of UHPC-CA. 

Furthermore, due to the distinction in the physical and chemical properties, the effect degrees of various supplementary cementitious materials on the strength of UHPC-CA are different, as shown in Figure 13. At the optimal mass ratios between the admixtures and cements (0.15 for slag, 0.2 for SF, and 0.1 for FA), the CS of UHPC-CA increased by 7.2%, 10%, and 5%, respectively, compared to the UHPC-CA without the admixture. This is because the SF with higher pozzolanic activity and smaller particle sizes contributes to a more significant micro-aggregate effect and pozzolanic effect. However, the larger specific surface area of SF leads to an increase in water demand. Therefore, it can be seen that when reaching a certain content of SF, the strength of UHPC-CA decreased with the increase in the mass ratio between SF and the cement due to the deterioration of the workability. Limited by the reactive activity, it is reported that the earlier strength of the UHPC-CA mixing with FA and slag is relatively lower, but the flowability of UHPC-CA is improved due to the low water absorption and the smooth and dense surface characteristics [52]. For a similar reason to that of the UHPC-CA with SF as above, the CS of UHPC-CA mixed with FA and slag tended to be first enhanced and then decreased in the later stage with the increase in the mass ratios. 

### 4.5. The Influence of the Sand Rate

**Figure 15:**
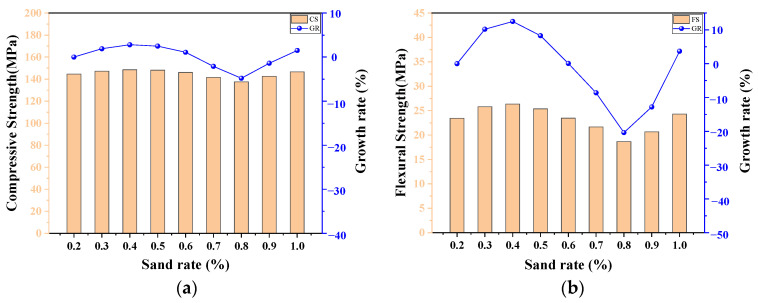
The influence of the sand rate on (**a**) compressive strength and (**b**) flexural strength.

Figure 15 shows the variations in the CS and FS of UHPC-CA with the sand rate. It can be observed that both the CS and FS of UHPC-CA exhibited a trend in first increasing and then decreasing as the sand rate varied from 20% to 100%. And, when the sand rate is about 40%, the strength of UHPC-CA is optimal. Zhang et al. [53] studied the effect of the sand rate on the mechanical properties of UHPC through ultrasonic testing, and the same variation tendency was observed. This indicates that there is a reasonable range in the sand rate for the proportions of UHPC-CA, and an excessive or low value in the sand rate may have an adverse effect on the mechanical properties of UHPC-CA. On the one hand, when the sand rate is less than the reasonable range, the slurry formed by sand, cementitious materials, and water is not sufficient to encapsulate the coarse aggregates, resulting in weakened bonding between the coarse aggregates and a decrease in the strength of the UHPC-CA. Meanwhile, the void between the coarse aggregates can not be filled effectively with a lack of fine aggregates, leading to an increase in the porosity within the UHPC-CA, and, consequently, a decrease in the strength of the UHPC-CA. On the other hand, when the sand rate exceeds the reasonable range, the specific surface area of the fine aggregates enlarges and the water demand increases accordingly, resulting in the decline of the workability of the UHPC-CA. Moreover, with the increase in the sand rate, the skeleton effect of the coarse aggregate is weakened, reducing the strength of the UHPC-CA. However, when the sand rate reaches 1.0, that is, when there is no coarse aggregate, both the CS and FS of UHPC-CA are improved because of the denser matrix. 

## 5. Conclusions

In this paper, the BP-ANN models for predicting the compressive strength (CS) and flexural strength (FS) of UHPC-CA were proposed based on a reliable dataset with a total of 220 valid samples taken from the experimental results in the studies published in the literature. Ten variables, including the contents of cement (C), silica fume (SF), slag, fly ash (FA), coarse aggregates (CA), steel fibers, the water–binder ratio (*w/b*), the sand rate (SR), the cement type (CT), and the curing method (CM), were set in the input layer, and a parametric study was conducted by the validated ANN model. The main conclusions can be drawn as below:

(1) Based on a process of trial and error, the BP-ANN models with 16 and 6 neurons in the hidden layer were selected as being optimal for predicting the CS and FS of UHPC-CA. The evaluated indices of the *R*^2^, *MSE*, *MAPE*, and *IAE* for the CS and FS prediction models were 0.91, 0.524, 0.023, and 0.018, and 0.96, 0.238, 0.08, and 0.064, respectively, demonstrating the higher accuracy and generality of the proposed ANN models.
(2) As the mass fraction of coarse aggregates in UHPC-CA increased from 0 to 60%, the CS of the UHPC-CA exhibited a trend of first increasing and then decreasing. The CS reached a maximum value when the coarse aggregate content reached 23%. While there were no significant variations in the FS of UHPC-CA when the mass fraction of coarse aggregate increased from 0 to 30%, it exhibited a sharp decline with the increase in CA. Consequently, it can be concluded that the optimum mass fraction of UHPC-CA is about 23%.
(3) When the water–binder ratio (*w*/*b*) increased from 0.10 to 0.28, both the CS and the FS of UHPC-CA presented a similar variation trend that was firstly improved (*w*/*b* = 0.1~1.18) and then decreased (*w*/*b* = 0.18~0.28). Meanwhile, it can be observed from the parametric analysis based on the ANN model that the addition of supplementary cementitious materials was beneficial to the strength of UHPC-CA within an appropriate range of the mass ratio between the admixtures and cements.
(4) The incorporation of steel fiber is favorable for both the CS and the FS of UHPC-CA. However, when the steel fiber volume fraction increases to a certain value, the workability of UHPC-CA may be worse due to the fiber agglomeration, and the defects in the microstructure will be increased leading to a reduction in the growth rate of the CS and FS of UHPC-CA. The parametric analysis indicated that the optimal steel fiber volume fraction for UHPC-CA is about 1.5~2%.
(5) An excessive or low value in the sand rate may have an adverse effect on the mechanical properties of UHPC-CA. Based on the predicted results from the ANN models, the FS and CS of UHPC-CA are the best when the sand rate is about 40%.

In summary, the proposed ANN model in this study can be used to determine the optimal mix proportion and predict the strength of UHPC-CA. Nevertheless, the local invariance of some input parameters may lead to slightly negative effects on the accuracy and modeling capability of the developed algorithms. Therefore, further experimental research is needed to expand the database and thereby increase the accuracy and applicability of the models.

## Figures and Tables

**Table 1:** Empirical formulas of compressive and flexural strengths of UHPC.

Author	Empirical Formula	Type of UHPC	Year
Xu et al. [13]	$f_{c}=10.78\frac{B}{W}-9.78\frac{C}{W}+102.33$	UHPC (without steel fiber)	2015
Savino et al. [14]	$f_{c}=75.1436+0.0499L_{f}/d_{f}+4.4016V_{f}$	UHPC (with steel fiber)	2018
Teng et al. [15]	$f_{f}=f_{fm}\left(1-V_{f}\right)+a\varphi \frac{L_{f}}{d_{f}}V_{f}\tau$	UHPC (with steel fiber)	2020
Ashkezari et al. [16]	$f_{c}=73.5+37.14V_{f}-6.06V_{f}^{2}$ $f_{f}=10.6+2.37V_{f}+1.31V_{f}^{2}$	UHPC (with steel fiber)	2020
Yang et al. [17]	$f_{c}=0.1843\times \left(6.1975X+0.1207Y\right)f_{ce,s}\times \left(\frac{B}{W}+9.629\right)$	UHPC (with steel fiber)	2021

Note: *f_c_*—compressive strength, *f_f_*—flexural strength, *f_ce_*—actual strength of cement, *B/W*—binder–water ratio, *C/W*—cement-water ratio, *V_f_*—fiber volume fraction, *L_f_*—fiber length, *d_f_*—fiber diameter, *f_fm_*—flexural strength of non-fiber UHPC mortar, *φ*—the fiber orientation coefficient, *τ*—the bond strength between UHPC matrix and fibers, *X*—the ratio of silica fume quality to mortar quality, *Y*—ratio of the sum of fly ash and slag to the total mass of cementitious materials, *f_ce,s_*—strength of mortar.

**Table 2:** ANN models for predicting mechanical properties of various types of concrete.

Year	Author	Database	No. of Input Neurons	No. of Hidden Layers	Concrete Type	Performance Metrics
2009	Prasad et al. [24]	300	10	2	HPC	*R*^2^ = 0.84~0.91
2009	Bilim et al. [28]	45	6	1	OPC	*R*^2^ = 0.92~0.95
2009	Sandemir et al. [29]	284	5	2	OPC	*R*^2^ = 0.996 *RMSE* = 1.036 *MAPE* = 2.433
2012	Khan et al. [30]	128	8	1	HPC	*R*^2^ = 0.93~0.95
2020	Kandiri et al. [31]	624	7	2	OPC	*RMSE* = 2.12~3.7 *MAPE* = 6.2~11.1 *R* = 0.93~0.98
2021	Congro et al. [32]	400	3	1	FRC	*R*^2^ = 0.92~0.94
2021	Moradi et al. [33]	134	8	1	CCM	*MSE* = 0.0067 *MAPE* = 20.3 *R* = 0.91

Note: HPC—High-performance concrete, OPC—Ordinary Portland concrete, FRC—fiber-reinforced concrete, CCM—concrete containing metakaolin.

**Table 3:** Statistical parameters of the UHPC-CA compressive strength datasets.

Attribute	Unit	Min	Max	Average	Standard Deviation	Q1	Q2	Q3	Mode
Cement	kg/m^3^	418.6	1058.8	773.7	174.7	638.52	805.28	894.63	894.63
Silica fume	kg/m^3^	37	308.7	173.53	72.1	125.25	166.64	220.89	125.25
Slag	kg/m^3^	0	467.9	100.95	128.24	0	0	163.74	0
Fly ash	kg/m^3^	0	260.6	73.14	76.69	0	53.68	133.14	0
Coarse aggregate	kg/m^3^	0	1348.8	398.6	433.8	0	309.29	837.07	0
Steel fiber	%	0	4	1.12	0.97	0.165	1	2	2
*W/B*	--	0.13	0.23	0.17	0.03	0.16	0.17	0.2	0.2
Sand rate	--	0.17	1	0.73	0.27	0.5	0.7	1	1
Compressive Strength	MPa	100	190	141	22.71	123.18	135	159.48	120

Note: The Q1, Q2, and Q3 are the first quartiles, medians, and third quartiles.

**Table 4:** Statistical parameters of the UHPC-CA flexural strength datasets.

Attribute	Unit	Min	Max	Average	Standard Deviation	Q1	Q2	Q3	Mode
Cement	kg/m^3^	436.6	1000.7	764.8	148.9	671.44	805.28	894.63	894.63
Silica fume	kg/m^3^	37	264	157.4	61.26	125.25	158.23	204.67	125.25
Slag	kg/m^3^	0	467.9	141.2	134.8	0	125.25	198.36	0
Fly ash	kg/m^3^	0	260.6	81.6	73.56	0	78.17	121.09	0
Coarse aggregate	kg/m^3^	0	1252.7	393.4	391.4	0	344.9	664.47	0
Steel fiber	%	0	4	1.26	0.94	0.24	1.5	2	2
*W/B*	--	0.13	0.23	0.17	0.03	0.15	0.16	0.2	0.2
Sand rate	--	0.17	1	0.72	0.25	0.5	0.7	1	1
Flexural Strength	MPa	5.36	43	23.01	9.26	18.18	23.3	28.18	23

Note: The Q1, Q2, and Q3 are the first quartiles, medians, and third quartiles.

## Data Availability

Data are contained within the article.

## Appendix A

**Table A1:** Experimental datasets for the CS of UHPC-CA collected from studies in the literature.

No.	Ref.	Country	C (kg/m^3^)	SF (kg/m^3^)	Slag (kg/m^3^)	FA (kg/m^3^)	CA (kg/m^3^)	V_f_ (%)	*W/B*	SR (%)	CM	CT	CS (MPa)
1	[4]	China	894.6	125.2	125.2	53.7	0	0	0.2	100	SC	P.O 42.5	120
2			894.6	125.2	125.2	53.7	0	0.5	0.2	100	SC	P.O 42.5	121
3			894.6	125.2	125.2	53.7	0	1	0.2	100	SC	P.O 42.5	120
4			894.6	125.2	125.2	53.7	0	1.5	0.2	100	SC	P.O 42.5	119.5
5			894.6	125.2	125.2	53.7	0	2	0.2	100	SC	P.O 42.5	114
6			894.6	125.2	125.2	53.7	0	2.5	0.2	100	SC	P.O 42.5	108
7			894.6	125.2	125.2	53.7	0	0.5	0.2	100	SC	P.O 42.5	127.5
8			894.6	125.2	125.2	53.7	0	1	0.2	100	SC	P.O 42.5	120
9			894.6	125.2	125.2	53.7	0	1.5	0.2	100	SC	P.O 42.5	120
10			894.6	125.2	125.2	53.7	0	2	0.2	100	SC	P.O 42.5	115
11			894.6	125.2	125.2	53.7	0	2.5	0.2	100	SC	P.O 42.5	108
12			894.6	125.2	125.2	53.7	0	0.5	0.2	100	SC	P.O 42.5	127.5
13			894.6	125.2	125.2	53.7	0	1	0.2	100	SC	P.O 42.5	123
14			894.6	125.2	125.2	53.7	0	1.5	0.2	100	SC	P.O 42.5	123
15			894.6	125.2	125.2	53.7	0	2	0.2	100	SC	P.O 42.5	120
16			894.6	125.2	125.2	53.7	0	2.5	0.2	100	SC	P.O 42.5	109
17	[30]	China	724.0	48.5	0	195.5	615.4	0	0.2	60	SC	P.O 52.5	144
18			706.6	47.3	0	190.8	939.8	0	0.2	40	SC	P.O 52.5	138
19			707.4	47.4	0	191.0	1252.1	0	0.2	20	SC	P.O 52.5	132
20			671.2	45.0	0	181.2	892.7	0	0.2	45	SC	P.O 52.5	137
21			634.0	42.5	0	171.2	841.4	0	0.2	50	SC	P.O 52.5	143
22			586.4	39.3	0	158.3	809.2	0	0.2	54	SC	P.O 52.5	136.25
23			724.1	48.5	0	195.5	1252.7	0	0.23	17	SC	P.O 52.5	100
24			677.3	45.4	0	182.9	1205.5	0	0.23	24	SC	P.O 52.5	113
25			636.1	42.6	0	171.8	1157.7	0	0.23	30	SC	P.O 52.5	120
26			593.3	39.8	0	160.2	1103.6	0	0.23	36	SC	P.O 52.5	135
27			556.7	37.3	0	150.3	1052.8	0	0.23	41	SC	P.O 52.5	141
28			552.8	37.0	0	149.2	895.5	0	0.23	50	SC	P.O 52.5	126
29	[31]	Japan	1058.8	264.7	0	0	0	0	0.16	100	SC	P.O 42.5	150.64
30			1058.8	264.7	0	0	0	0	0.16	100	SC	P.O 42.5	129.6
31			1058.8	264.7	0	0	0	0	0.16	100	SC	P.O 42.5	121.25
32			1058.8	264.7	0	0	0	0	0.16	100	SC	P.O 42.5	130.1
33			1058.8	264.7	0	0	0	0	0.16	100	SC	P.O 42.5	133.3
34			1018.9	254.7	0	0	0	0	0.16	100	SC	P.O 42.5	149.75
35			1018.9	254.7	0	0	0	0	0.16	100	SC	P.O 42.5	153.1
36			1018.9	254.7	0	0	0	0	0.16	100	SC	P.O 42.5	131.8
37			885.2	221.3	0	0	0	0	0.16	100	SC	P.O 42.5	126.13
38			934.3	233.6	0	0	0	0	0.16	100	SC	P.O 42.5	139.67
39			989.0	247.3	0	0	0	0	0.16	100	SC	P.O 42.5	156.87
40			1050.6	262.6	0	0	0	0	0.16	100	SC	P.O 42.5	142.4
41	[32]	China	935.5	308.7	0	0	0	0	0.17	100	SC	P.O 42.5	105
42			935.5	308.7	0	0	0	1	0.17	100	SC	P.O 42.5	130
43			935.5	308.7	0	0	0	2	0.17	100	SC	P.O 42.5	148
44			935.5	308.7	0	0	0	3	0.17	100	SC	P.O 42.5	160
45			935.5	308.7	0	0	0	1	0.17	100	SC	P.O 42.5	131
46			935.5	308.7	0	0	0	2	0.17	100	SC	P.O 42.5	150
47			935.5	308.7	0	0	0	3	0.17	100	SC	P.O 42.5	162
48			935.5	308.7	0	0	0	1	0.17	100	SC	P.O 42.5	145
49			935.5	308.7	0	0	0	2	0.17	100	SC	P.O 42.5	160
50			935.5	308.7	0	0	0	3	0.17	100	SC	P.O 42.5	167
51	[33]	China	767.3	184.1	0	237.9	0	0.24	0.18	100	SC	P.O 52.5	105
52			630.3	145.0	0	195.4	466.4	0.28	0.18	70	SC	P.O 52.5	125
53			515.5	118.6	0	159.8	881.6	0.33	0.18	50	SC	P.O 52.5	135
54			430.8	99.1	0	133.6	1150.3	0.39	0.18	40	SC	P.O 52.5	125
55			615.9	141.7	0	190.9	474.2	0.28	0.18	70	SC	P.O 52.5	122
56			502.1	115.5	0	155.7	893.8	0.33	0.18	50	SC	P.O 52.5	126
57			418.6	96.3	0	129.8	1163.6	0.39	0.18	40	SC	P.O 52.5	125
58			640.2	147.3	0	198.5	461.0	0.28	0.18	70	SC	P.O 52.5	119
59			523.5	120.4	0	162.3	874.3	0.33	0.18	50	SC	P.O 52.5	122
60			437.8	100.7	0	135.7	1142.7	0.39	0.18	40	SC	P.O 52.5	119
61			572.4	131.6	0	177.4	498.0	0.28	0.18	70	SC	P.O 52.5	105
62			512.6	117.9	0	158.9	1061.1	0.33	0.18	40	SC	P.O 52.5	117
63			425.5	97.9	0	131.9	1348.8	0.39	0.18	30	SC	P.O 52.5	115
64	[34]	Britain	731.1	131.6	467.9	0.0	0	2	0.15	100	SC	P.O 42.5	150.56
65			731.1	131.6	467.9	0.0	0	0	0.15	100	SC	P.O 42.5	121.32
66	[1]	China	998.3	219.6	429.3	0.0	0	2	0.13	100	HTC	P.O 52.5	169.3
67			887.3	195.2	381.5	0.0	292.8	2	0.13	72	HTC	P.O 52.5	171.4
68			846.7	186.3	364.1	0.0	414.9	2	0.13	63	HTC	P.O 52.5	175.1
69			805.3	177.2	346.3	0.0	515.4	2	0.13	57	HTC	P.O 52.5	169.6
70			805.3	177.2	346.3	0.0	515.4	2	0.13	57	HTC	P.O 52.5	172.5
71			763.1	167.9	328.2	0.0	625.8	2	0.13	51	HTC	P.O 52.5	168.8
72			805.3	177.2	346.3	0.0	515.4	2	0.13	57	HTC	P.O 52.5	183.4
73			805.3	177.2	346.3	0.0	515.4	2	0.13	57	HTC	P.O 52.5	189.4
74	[35]	China	673.0	215.4	215.4	121.1	0	0.21	0.13	100	SC	P.O 42.5	126
75			594.1	190.1	190.1	106.9	0	0.23	0.13	100	SC	P.O 42.5	127
76			530.7	169.8	169.8	95.5	0	0.25	0.13	100	SC	P.O 42.5	128
77			478.6	153.2	153.2	86.2	0	0.27	0.13	100	SC	P.O 42.5	130.5
78			436.6	139.7	139.7	78.6	0	0.28	0.13	100	SC	P.O 42.5	129
79			595.4	190.5	190.5	107.2	160.7	0.23	0.2	80	SC	P.O 42.5	134
80			510.3	163.3	163.3	91.8	280.6	0.25	0.13	70	SC	P.O 42.5	135.5
81			467.0	149.5	149.5	84.1	383.0	0.26	0.13	60	SC	P.O 42.5	136.3
82			436.6	139.7	139.7	78.6	475.9	0.28	0.13	50	SC	P.O 42.5	134.5
83	[2]	China	796.2	246.8	0.0	183.1	0.0	1.28	0.15	100	HTC	P.O 42.5	124.5
84			851.5	264.0	0.0	195.8	119.2	1.28	0.15	90	HTC	P.O 42.5	125
85			590.7	183.1	0.0	135.9	165.4	1.28	0.15	90	HTC	P.O 42.5	127.5
86			688.6	213.5	0.0	158.4	296.1	1.28	0.15	80	HTC	P.O 42.5	128.5
87			735.5	228.0	0.0	169.2	419.2	1.28	0.15	70	HTC	P.O 42.5	126
88			652.5	202.3	0.0	150.1	463.3	1.28	0.15	70	HTC	P.O 42.5	122
89	[36]	China	521.2	260.6	0.0	260.6	0	0	0.19	100	HTC	P.O 42.5	103.9
90			521.2	260.6	0.0	260.6	0	1	0.19	100	HTC	P.O 42.5	116.7
91			521.2	260.6	0.0	260.6	0	1.5	0.19	100	HTC	P.O 42.5	120.2
92			521.2	260.6	0.0	260.6	0	2	0.19	100	HTC	P.O 42.5	123.7
93	[37]	China	769.5	123.1	0.0	0.0	977.3	0	0.2	40	SC	P.O 52.5	110
94			816.0	130.6	0.0	0.0	938.4	1	0.2	40	SC	P.O 52.5	140
95			868.5	139.0	0.0	0.0	894.6	2	0.2	40	SC	P.O 52.5	152
96			916.3	137.4	0.0	0.0	861.3	3	0.2	40	SC	P.O 52.5	163
97			969.6	135.7	0.0	0.0	824.2	4	0.2	40	SC	P.O 52.5	167
98			871.9	130.8	0.0	0.0	898.1	2	0.2	40	SC	P.O 52.5	158
99			871.9	130.8	0.0	0.0	898.1	2	0.2	40	SC	P.O 52.5	160
100			871.9	130.8	0.0	0.0	898.1	2	0.2	40	SC	P.O 52.5	159.5
101			871.9	130.8	0.0	0.0	898.1	2	0.2	40	SC	P.O 52.5	145
102			871.9	130.8	0.0	0.0	898.1	2	0.2	40	SC	P.O 52.5	139
103	[38]	China	1000.7	250.2	200.1	100.1	0.0	2	0.16	100	HTC	P.O 42.5	173.4
104			931.7	232.9	186.3	93.2	205.0	2	0.16	80	HTC	P.O 42.5	177.4
105			818.7	204.7	163.7	81.9	368.4	2	0.16	70	HTC	P.O 42.5	180.1
106			777.4	194.4	155.5	77.7	520.9	2	0.16	60	HTC	P.O 42.5	178.2
107			750.4	187.6	150.1	75.0	675.4	2	0.16	50	HTC	P.O 42.5	167.3
108			818.7	204.7	163.7	81.9	491.2	2	0.16	60	HTC	P.O 42.5	151.9
109			862.3	215.6	172.5	86.2	344.9	2	0.16	70	HTC	P.O 42.5	175.3
110			862.3	215.6	172.5	86.2	344.9	2	0.16	70	HTC	P.O 42.5	173.1
111			862.3	215.6	172.5	86.2	344.9	2	0.16	70	HTC	P.O 42.5	178.7
112			862.3	215.6	172.5	86.2	344.9	2	0.16	70	HTC	P.O 42.5	181.2
113			965.0	241.2	193.0	96.5	193.0	2	0.16	80	HTC	P.O 42.5	176.3
114			1000.7	250.2	200.1	100.1	0	2	0.16	100	HTC	P.O 42.5	172.5
115	[39]	China	998.3	219.6	429.3	0	0	2	0.13	100	SC	P.O 52.5	153.6
116			871.6	191.7	374.8	0	322.5	2	0.13	70	SC	P.O 52.5	160.6
117			833.5	183.4	358.4	0	458.4	2	0.13	60	SC	P.O 52.5	156.7
118			757.5	166.6	325.7	0	515.1	2	0.13	60	SC	P.O 52.5	159.4
119			757.5	166.6	325.7	0	515.1	2.5	0.13	60	SC	P.O 52.5	154.1
120			757.5	166.6	325.7	0	515.1	1	0.13	60	SC	P.O 52.5	149.4
121			770.4	169.5	331.3	0	631.8	2	0.13	50	SC	P.O 52.5	148.6
122	[40]	China	600.0	120.0	78.0	0	1242.0	2	0.16	30	HTC	P.O 42.5	175
123			638.5	127.7	83.0	0	1200.4	2	0.16	30	HTC	P.O 42.5	180
124			679.9	136.0	88.4	0	1155.8	2	0.16	30	HTC	P.O 42.5	185
125			638.5	127.7	83.0	0	1200.4	2	0.16	30	HTC	P.O 42.5	190
126			638.5	127.7	83.0	0	1200.4	2	0.16	30	HTC	P.O 42.5	187
127	[41]	China	671.4	214.9	214.9	120.9	0	0.15	0.16	100	SC	P.O 42.5	132
128			671.4	214.9	214.9	120.9	0	0.15	0.16	100	SC	P.O 42.5	129
129			671.9	215.0	215.0	121.0	551.0	0.15	0.16	57	SC	P.O 42.5	135
130			471.9	151.0	151.0	84.9	901.4	0.15	0.2	46	SC	P.O 42.5	138

Note: C—Cement; SF—Silica fume; FA—Fly ash; CA—Coarse aggregate; V_f_—Steel fiber volume fraction; *W/B*—Water–binder ratio; SR—Sand rate; CM—Curing method; CT—Cement type.

**Table A2:** Experimental datasets for the FS of UHPC-CA collected from studies in the literature.

No.	Ref.	Country	C (kg/m^3^)	SF (kg/m^3^)	Slag (kg/m^3^)	FA (kg/m^3^)	CA (kg/m^3^)	V_f_ (%)	*W/B*	SR (%)	CM	CT	FS (MPa)
1	[4]	China	894.6	125.2	125.2	53.7	0.0	0	0.2	100	SC	P.O 42.5	20
2			894.6	125.2	125.2	53.7	0.0	0.5	0.2	100	SC	P.O 42.5	20.5
3			894.6	125.2	125.2	53.7	0.0	1	0.2	100	SC	P.O 42.5	21.8
4			894.6	125.2	125.2	53.7	0.0	1.5	0.2	100	SC	P.O 42.5	22.2
5			894.6	125.2	125.2	53.7	0.0	2	0.2	100	SC	P.O 42.5	23
6			894.6	125.2	125.2	53.7	0.0	2.5	0.2	100	SC	P.O 42.5	24
7			894.6	125.2	125.2	53.7	0.0	0.5	0.2	100	SC	P.O 42.5	20.2
8			894.6	125.2	125.2	53.7	0.0	1	0.2	100	SC	P.O 42.5	22
9			894.6	125.2	125.2	53.7	0.0	1.5	0.2	100	SC	P.O 42.5	23
10			894.6	125.2	125.2	53.7	0.0	2	0.2	100	SC	P.O 42.5	24
11			894.6	125.2	125.2	53.7	0.0	2.5	0.2	100	SC	P.O 42.5	24.5
12			894.6	125.2	125.2	53.7	0.0	0.5	0.2	100	SC	P.O 42.5	23
13			894.6	125.2	125.2	53.7	0.0	1	0.2	100	SC	P.O 42.5	23.5
14			894.6	125.2	125.2	53.7	0.0	1.5	0.2	100	SC	P.O 42.5	24
15			894.6	125.2	125.2	53.7	0.0	2	0.2	100	SC	P.O 42.5	24.2
16			894.6	125.2	125.2	53.7	0.0	2.5	0.2	100	SC	P.O 42.5	25
17	[30]	China	724.0	48.5	0.0	195.5	615.4	0	0.2	60	SC	P.O 52.5	8.5
18			706.6	47.3	0.0	190.8	939.8	0	0.2	40	SC	P.O 52.5	7.8
19			707.4	47.4	0.0	191.0	1252.1	0	0.2	20	SC	P.O 52.5	8
20			671.2	45.0	0.0	181.2	892.7	0	0.2	45	SC	P.O 52.5	9
21			634.0	42.5	0.0	171.2	841.4	0	0.2	50	SC	P.O 52.5	9
22			586.4	39.3	0.0	158.3	809.2	0	0.2	54	SC	P.O 52.5	8
23			724.1	48.5	0.0	195.5	1252.7	0	0.23	17	SC	P.O 52.5	5.5
24			677.3	45.4	0.0	182.9	1205.5	0	0.23	24	SC	P.O 52.5	6
25			636.1	42.6	0.0	171.8	1157.7	0	0.23	30	SC	P.O 52.5	7
26			593.3	39.8	0.0	160.2	1103.6	0	0.23	36	SC	P.O 52.5	7.5
27			556.7	37.3	0.0	150.3	1052.8	0	0.23	41	SC	P.O 52.5	7.5
28			552.8	37.0	0.0	149.2	895.5	0	0.23	50	SC	P.O 52.5	8
29	[34]	Britain	731.1	131.6	467.9	0.0	0.0	2	0.15	100	SC	P.O 42.5	9.07
30			731.1	131.6	467.9	0.0	0.0	0	0.15	100	SC	P.O 42.5	5.36
31	[1]	China	998.3	219.6	429.3	0.0	0.0	2	0.13	100	HTC	P.O 52.5	31.4
32			887.3	195.2	381.5	0.0	292.8	2	0.13	72	HTC	P.O 52.5	30.9
33			846.7	186.3	364.1	0.0	414.9	2	0.13	63	HTC	P.O 52.5	28.7
34			805.3	177.2	346.3	0.0	515.4	2	0.13	57	HTC	P.O 52.5	24.5
35			805.3	177.2	346.3	0.0	515.4	2	0.13	57	HTC	P.O 52.5	26.3
36			763.1	167.9	328.2	0.0	625.8	2	0.13	51	HTC	P.O 52.5	22.1
37			805.3	177.2	346.3	0.0	515.4	2	0.13	57	HTC	P.O 52.5	28.3
38			805.3	177.2	346.3	0.0	515.4	2	0.13	57	HTC	P.O 52.5	29.7
39	[35]	China	673.0	215.4	215.4	121.1	0.0	0.21	0.13	100	SC	P.O 42.5	26.2
40			594.1	190.1	190.1	106.9	0.0	0.23	0.13	100	SC	P.O 42.5	26.7
41			530.7	169.8	169.8	95.5	0.0	0.25	0.15	100	SC	P.O 42.5	26.4
42			478.6	153.2	153.2	86.2	0.0	0.27	0.15	100	SC	P.O 42.5	26
43			436.6	139.7	139.7	78.6	0.0	0.28	0.13	100	SC	P.O 42.5	23.5
44			595.4	190.5	190.5	107.2	160.7	0.23	0.13	80	SC	P.O 42.5	27.5
45			510.3	163.3	163.3	91.8	280.6	0.25	0.23	70	SC	P.O 42.5	27.1
46			467.0	149.5	149.5	84.1	383.0	0.26	0.13	60	SC	P.O 42.5	25.4
47			436.6	139.7	139.7	78.6	475.9	0.28	0.15	50	SC	P.O 42.5	20.9
48	[2]	China	796.2	246.8	0.0	183.1	0.0	1.28	0.15	100	HTC	P.O 42.5	18.4
49			851.5	264.0	0.0	195.8	119.2	1.28	0.15	90	HTC	P.O 42.5	19.5
50			590.7	183.1	0.0	135.9	165.4	1.28	0.15	90	HTC	P.O 42.5	18.6
51			688.6	213.5	0.0	158.4	296.1	1.28	0.15	80	HTC	P.O 42.5	18.7
52			735.5	228.0	0.0	169.2	419.2	1.28	0.15	70	HTC	P.O 42.5	19.3
53			652.5	202.3	0.0	150.1	463.3	1.28	0.15	70	HTC	P.O 42.5	18.1
54	[36]	China	521.2	260.6	0.0	260.6	0.0	0	0.19	100	HTC	P.O 42.5	10.5
55			521.2	260.6	0.0	260.6	0.0	1	0.19	100	HTC	P.O 42.5	16.7
56			521.2	260.6	0.0	260.6	0.0	1.5	0.19	100	HTC	P.O 42.5	23.1
57			521.2	260.6	0.0	260.6	0.0	2	0.19	100	HTC	P.O 42.5	23.8
58	[37]	China	769.5	123.1	0.0	0.0	977.3	0	0.2	40	SC	P.O 52.5	23
59			816.0	130.6	0.0	0.0	938.4	1	0.2	40	SC	P.O 52.5	24
60			868.5	139.0	0.0	0.0	894.6	2	0.2	40	SC	P.O 52.5	37
61			916.3	137.4	0.0	0.0	861.3	3	0.2	40	SC	P.O 52.5	42
62			969.6	135.7	0.0	0.0	824.2	4	0.2	40	SC	P.O 52.5	35
63			871.9	130.8	0.0	0.0	898.1	2	0.2	40	SC	P.O 52.5	40
64			871.9	130.8	0.0	0.0	898.1	2	0.2	40	SC	P.O 52.5	40
65			871.9	130.8	0.0	0.0	898.1	2	0.2	40	SC	P.O 52.5	43
66			871.9	130.8	0.0	0.0	898.1	2	0.2	40	SC	P.O 52.5	40
67			871.9	130.8	0.0	0.0	898.1	2	0.2	40	SC	P.O 52.5	42
68	[38]	China	1000.7	250.2	200.1	100.1	0.0	2	0.16	100	HTC	P.O 42.5	36.25
69			931.7	232.9	186.3	93.2	205.0	2	0.16	80	HTC	P.O 42.5	36.45
70			818.7	204.7	163.7	81.9	368.4	2	0.16	70	HTC	P.O 42.5	36.34
71			777.4	194.4	155.5	77.7	520.9	2	0.16	60	HTC	P.O 42.5	32.22
72			750.4	187.6	150.1	75.0	675.4	2	0.16	50	HTC	P.O 42.5	29.7
73			818.7	204.7	163.7	81.9	491.2	2	0.16	60	HTC	P.O 42.5	22.75
74			862.3	215.6	172.5	86.2	344.9	2	0.16	70	HTC	P.O 42.5	25.7
75			862.3	215.6	172.5	86.2	344.9	2	0.16	70	HTC	P.O 42.5	27.8
76			862.3	215.6	172.5	86.2	344.9	2	0.16	70	HTC	P.O 42.5	31.4
77			862.3	215.6	172.5	86.2	344.9	2	0.16	70	HTC	P.O 42.5	34.1
78			965.0	241.2	193.0	96.5	193.0	2	0.16	80	HTC	P.O 42.5	28.7
79			1000.7	250.2	200.1	100.1	0.0	2	0.16	100	HTC	P.O 42.5	32.2
80	[39]	China	998.3	219.6	429.3	0.0	0.0	2	0.13	100	SC	P.O 52.5	20.8
81			871.6	191.7	374.8	0.0	322.5	2	0.13	70	SC	P.O 52.5	18
82			833.5	183.4	358.4	0.0	458.4	2	0.13	60	SC	P.O 52.5	17.8
83			757.5	166.6	325.7	0.0	515.1	2	0.13	60	SC	P.O 52.5	17.1
84			757.5	166.6	325.7	0.0	515.1	2.5	0.13	60	SC	P.O 52.5	17.5
85			757.5	166.6	325.7	0.0	515.1	1	0.13	60	SC	P.O 52.5	19.5
86			770.4	169.5	331.3	0.0	631.8	2	0.13	50	SC	P.O 52.5	16.4
87	[41]	China	671.4	214.9	214.9	120.9	0.0	0.15	0.16	100	SC	P.O 42.5	29.42
88			671.4	214.9	214.9	120.9	0.0	0.15	0.16	100	SC	P.O 42.5	26.36
89			671.9	215.0	215.0	121.0	551.0	0.15	0.16	57	SC	P.O 42.5	22.37
90			471.9	151.0	151.0	84.9	901.4	0.15	0.2	46	SC	P.O 42.5	16.86

Note: C—Cement; SF—Silica fume; FA—Fly ash; CA—Coarse aggregate; V_f_—Steel fiber volume fraction; *W/B*—Water–binder ratio; SR—Sand rate; CM—Curing method; CT—Cement type.
